# Use of human fat grafting in the prevention of perineural adherence: Experimental study in athymic mouse

**DOI:** 10.1371/journal.pone.0176393

**Published:** 2017-04-26

**Authors:** Mario Cherubino, Igor Pellegatta, Alessandro Crosio, Luigi Valdatta, Stefano Geuna, Rosalba Gornati, Pierluigi Tos

**Affiliations:** 1 Department of Biotechnologies and Life Sciences, University of Insubria, Varese, Italy; 2 Reconstructive Microsurgery Unit, Traumatology Department, CTO Hospital, Turin, Italy; 3 Human Anatomy Laboratory, Clinical and Biological Sciences Department, University of Turin, AOU San Luigi Gonzaga, Orbassano, Italy; 4 Department of Orthopedic, Traumatologic and Hand Surgery, ASST Gaetano Pini, Milan, Italy; Szegedi Tudomanyegyetem, HUNGARY

## Abstract

Perineural adherences represent a problem after surgery involving peripheral neural system. Fat-grafting with adipose derived stem cells (ASCs) with their pro-regenerative characteristics can be important to prevent the neural damage or to facilitate the neural regeneration. Our idea was to use the fat-grafting as an anti-adherence device and test its efficacy on a postsurgical scar animal model and comparing to an antiadhesive gel. 32 athymic mice were operated under magnification, we exposed both sciatic nerves. We randomly divided all sciatic nerves into four experimental groups: burning (1), burning + carboxy-methylcellulose and poly- ethylene oxide (CMC-PEO) (2) + human adipose fat tissue (3), control group (4). Bio-mechanical evaluation was performed to measure the peak force required to pull out the nerve from the muscular bed. Results: in the CMC-PEO group the peak pull out force was 0.37 Newton. In the fat grafted group we registered a peak pull out force of 0.35 N (t Student 0.913). In burning group the force necessary to tear the nerve apart was markedly superior (0.46 N). In control group, we reported the minimal strength (0.31 N) to slide the nerve from the tissue. Histologically, in the group treated with fat-grating, a thinner scar layer was highlighted. Considering the results of this study we can support the efficacy in animal experimental model of fat graft as an anti-adherence device in peripheral nerve surgery.

## Introduction

Perineural adherences can become a major problem after any surgery involving the peripheral neural system (PNS). Entrapment syndrome can lead to clinically important symptomatology, as persistent pain, that can compromise the quality of life of the patient [1]. In the PNS, perineural fibrosis is the second most frequent cause of recurrent carpal tunnel syndrome (CTS). Patients with recurrent CTS often must undergo reoperation owing to debilitating symptoms that affect daily activity. The most frequent pathologies connected with this condition are traction neuropathies and type II Complex Regional Pain Syndrome.

In the last 15 years, numerous anti-adherence devices [2] have been developed to prevent the physiologic development of perineural fibrosis after a surgical trauma. Products such as Dynavisc^®^ [3], ADCON^®^ [4, 5] and Hyaloglyde^®^ [6, 7] are broadly used in surgeries involving manipulation of tendons and nerves in the neural central system. Few reports are available in the literature regarding the efficacy of these types of barriers in the peripheral neural system. A previous study conducted by our team recognized the utility of one of these barriers in the prevention of perineural adherence in mouse models [8]. However, the main drawback concerning these devices are their high cost.

Fat grafting properties are well known and have become more popular in last few years [9, 10]. Adipose-derived stem cells (ASCs) and other cells included in the stromal vascular fraction (SVF) survive with the lipografting (or fat grafting) technique. They donate a pro-regenerative pattern of characteristics that can be important for preventing neural damage or facilitating neural regeneration after a trauma [11].

A fat graft harvest with cannula as lipoaspirate, has a similar consistency compared with the anti-adherence devices that are available in the market. Some studies [12] report the use of fat grafting in the treatment of median nerve decompression in carpal tunnel syndrome [13] or in aponeurectomy in Dupuytren’s disease [14] to prevent new adherence formation.

In this study, we used a human lipoaspirated fat graft as an adhesion barrier in a sciatic nerve model in athymic mouse to prevent the scar formation and we compared with a carboxy-methylcellulose (CMC) with poly- ethylene oxide (PEO).

## Patients and methods

### Experimental surgery

All procedures were performed in accordance with the Local Ethical Committee and the European Communities Council Directive of EU/63/2010. The experimental study was approved by Italian Ministero della Salute (192/2013-A approved on July 25th 2013). A total forty-eight thighs of athymic mice have been analyzed (CD1 nude, 5 weeks old, average weight 28 g, *Charles River Laboratories*, *Calco*, *Lecco*, *Italy*). For each group, 16 nerves have been randomized (16 athymic mice in each experimental group).

**Fig 1:**
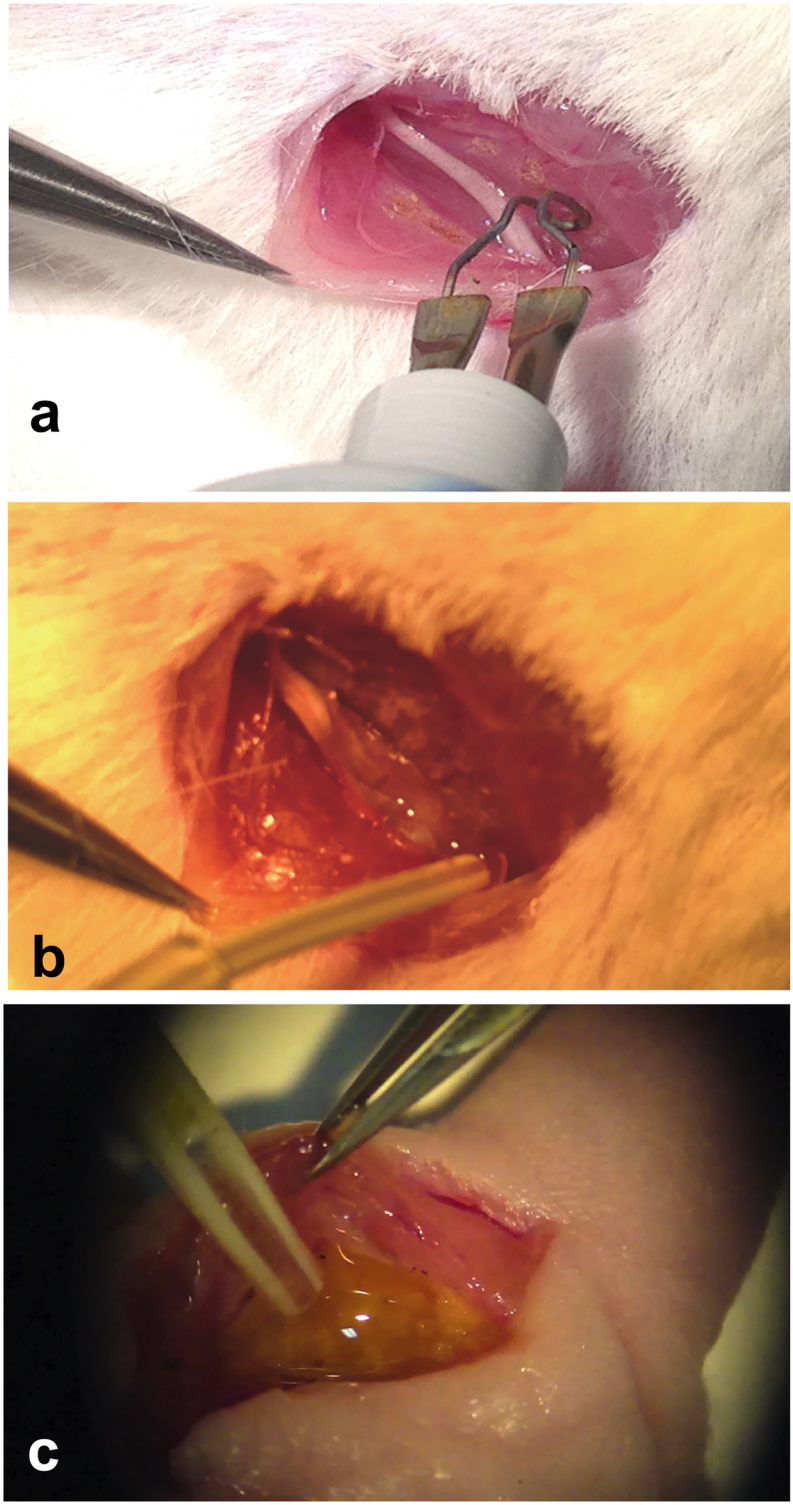
Surgical procedures on the sciatic nerve. After gluteal splitting exposure of the sciatic nerve, the surrounding muscles have been cauterized a. In panel b, about 0,5 ml of CMC-PEO gel was applied around the sciatic nerve. In c, 0.5 ml of human fat graft was applied around the nerve after a burning injury.

All animals were housed, following the directives of Our Institutional Council (OBPA—Organismo preposto al benessere animale) under standard light conditions with unlimited access to food and water. After the surgery the mouse was controlled on daily basis checking for mutilation or any unusual behavior. Analgesic post-op treatment was induced by Carprofen (4 mg/kg for 2–3 days). Fourteen nerves from each group were included in bio-mechanical analysis and two nerves per group underwent histological analysis. To perform our surgery on the animals, we employed a versatile, widespread and cost-effective animal model that has been described recently by our group in a previous paper [15]. In summary, the athymic adult mice were anaesthetized using a combination of 100 mg/kg of Ketamine and 15 mg/kg of Xylazine applied intraperitoneally. Under microscopic magnification, we exposed both sciatic nerves by a gluteal splitting incision, to clearly view the sciatic nerve from the gluteal vein to the trifurcation as presented in Fig 1a.

We Used the same procedure for the CMC-PEO group as described in our previous paper [8]

Accordingly, we randomly divided all sciatic nerves into 4 experimental groups: burning group (1), tissue burning + CMC-PEO (2), tissue burning + human adipose tissue (3), and control group (4). The CMC-PEO was analyzed comparing the results obtain in our previous study [8].

In the burning group, after retraction of the nerve, we burned the muscle surface with a surgical diathermy (*Bovie Cautery pen micro tip*, Bovie Medical Corporation, NY, USA) for approximately 0.8 cm along the nerve bed, to create the scar adhesion as previously described [15, 16].

In the burning + lipografting group, we burned the muscle surface exactly as the previous group and then we applied a thin layer of lipoaspirated and centrifuged human fat tissue (approximately 0.5 ml for thigh) on top of the muscular burnt bed, as displayed in Fig 1c. The amount used had created a thin layer of fat tissue around the nerve. We never overload with the fat, using always the same amount (0.5 ml), to let at the transplant fat tissue to survive.

In the CMC-PEO In the Burned 1 anti-adhesion gel group, after muscle burning as described above, we applied a small quantity of gel (1 ml) to the muscular bed to completely cover and surround the nerve as illustrated in Fig 1b.

In the control group, we exposed the sciatic nerve and immediately closed the skin with 3–0 Prolene sutures.

At the end of the follow up after the experimental surgery, we euthanized the animals in sealed chamber with carbon dioxide inhalation once the animal was unconscious after sedation with isoflurane.

### Fat grafting harvesting technique

The fat tissue was harvested the same morning of the experimental surgery on animals from one informed female human patient (age: 34. BMI: 25) who underwent liposuction for cosmetic reason. Written informed consent was obtained using an independent institutional approved protocol by University and Hospital Ethical Committees (Comitato Etico ASST Sette Laghi) in accordance with declaration of Helsinki. The patient was healthy and not smoker without any comorbidities. After infiltration with tumescent fluid the cells and the fat were harvested from the lateral thigh region with Khouri’s 2-mm cannula connect to a 10ml Syringe. The lipoaspirate was purified using a centrifuge (MPW 223e, MPW medical instruments, Poland) at 1200 rpm for 3 min in accordance with guidelines of Coleman [17]. The top layer of residual oil and the tumescent fluid was discarded before transferring the purified lipoaspirate to 1-mL syringes. The sample that was immediately conserved in ice and applied in the experimental animal in the next 90 minutes. The athymic mice were required to prevent a xenogeneic reaction to the injection of human fat but develop enough scar tissue around the nerve as demonstrated in our previous study [8]. In our other parallel study about ASCs properties [18] we analyzed the sub populations of lipoaspirate used in this study, showing a homogenous population of adipose-derived stem cells (ASCs) characterized by the presence of CD44, CD90, CD105 and HLA class I markers and the absence of the CD45 marker. These results are comparable to other studies available in the literature [19].

### Bio-mechanical evaluation method and sample extraction

**Fig 2:**
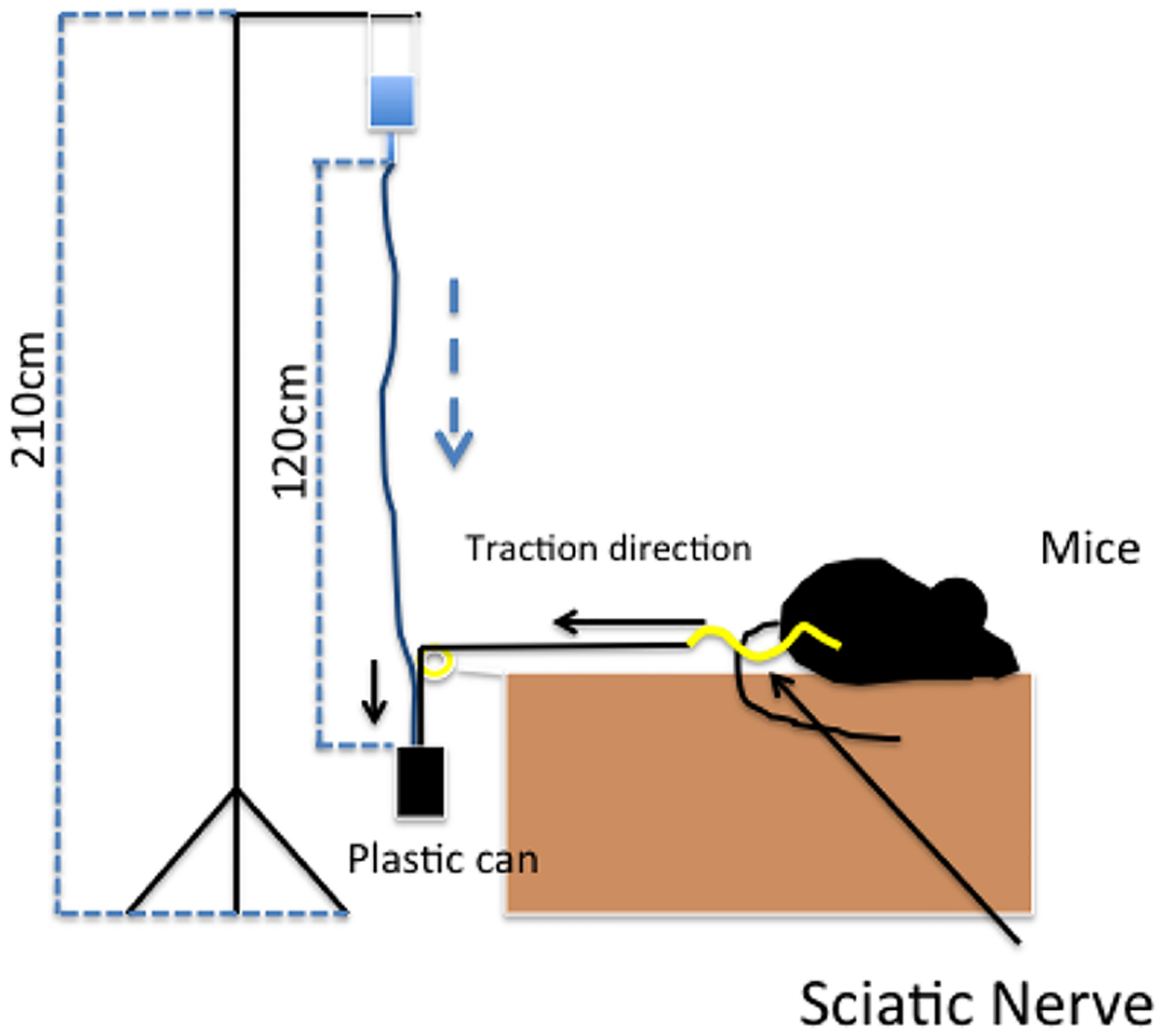
A schematic view of the extraction tool.

After four weeks, all animals were sacrificed by CO^2^ inhalation in a closed chamber. In each group, bio-mechanical evaluation was performed. Two nerves from each group were not tested bio-mechanically but were processed for histological evaluation. Bio-mechanical evaluation was performed to measure the peak force required to pull the nerve out from the muscular bed by methods and tools described in a previous work [15]. Briefly, the tool consists of using a force applied constantly to the nerve until the traction breaks the adhesions between the nerve and the surrounding tissue. The sciatic nerve, during biomechanical analysis harvesting, has been dissected from its origin at the foramen ischiadicum, then the proximal stump has been connected to the plastic can using a 9–0 knot. Distally the nerve was dissected at the trifurcation at the knee, then the sciatic nerve has been cut just above the trifurcation. This method had been studied and described in a previous work cited in references section [8][20] A schematic view of the extraction tool is presented in Fig 2. The force is reported in Newtons (N). A normality test was performed by the Kolmogorov Smirnov test. Statistical analysis of the results was performed using Student t-tests. Statistical significance was established when p < 0.05.

For histological analysis, the posterior space of the thigh with nerve and scar tissue inside the muscles was harvested “en-bloc”. The proximal end was marked with 9–0 Nylon under magnification. Nerves were fixed with 4% paraformaldehyde (Fulka) in PBS (Phosphate Buffered Saline) for 2–4 hours followed by washing in 0.2% glycine in PBS. After fixation, samples underwent dehydration in ethanol from 50% to 100%, followed by a dyaphanization step in xylol. Specimens were then maintained in liquid paraffin at 60°C overnight and then passed to a second step in liquid paraffin at 60°C before polymerization at room temperature. After paraffin embedding, transverse sections (10 μm thickness) were obtained and stained with Picrosirius staining (picric acid + Sirius red) according to a previously described protocol [21]. Sirius Red stains collagen I and III fibers. In histological sections, collagen appears in red. No statistical analysis was conducted on these samples.

## Results

No adverse effects or xenogeneic reactions were recorded. All animals survived four weeks after the surgical procedures. No wound leaks or pathological secretions were observed.

### Biomechanical analysis

**Fig 3:**
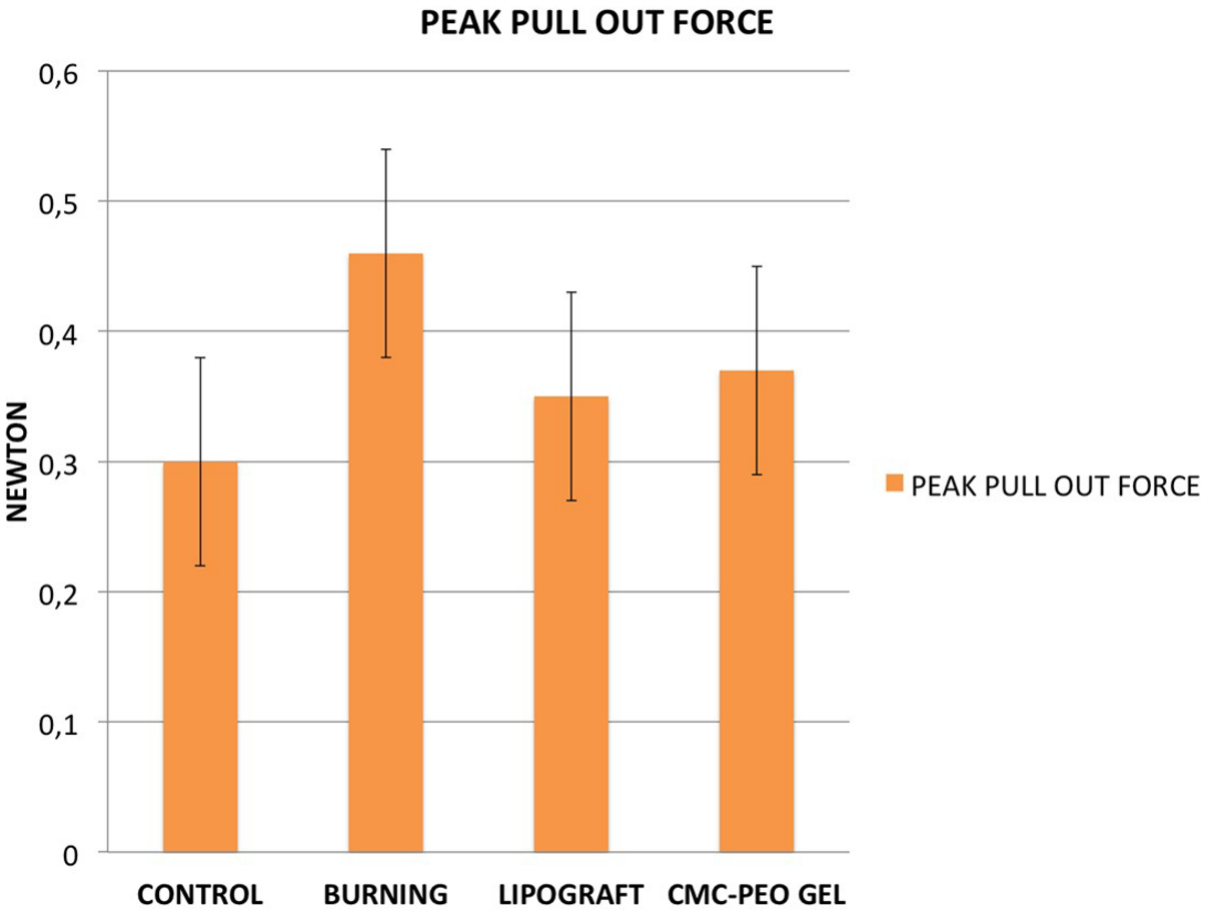
Mean and standard deviation of peak pull out force in Newton for each group.

Results of the bio-mechanical analysis are summarized in Table 1 as the mean force in Newtons (N) that is necessary to tear the nerve off the muscle. The control group shows a required mean force of 0.30 N, after a burning injury, the force increased to 0.46 N. On the group treated with lipografting, the force needed to break the scar tissue decreased to 0.35 N. Similar values in the CMC-PEO group 0.37 N. Each group underwent a Kolmogorov Smirnov test, showing that all four groups present a normal distribution (p > 0.15). The statistical analysis showed that the injury method creates valid scar tissue (burning vs control p < 0.001); additionally, a strong significant difference between the burned group and the lipografting group (p < 0.001) indicated that lipografting can reduce the scar tissue around a nerve after a surgical injury. No statistic difference has been observed between the lipografting group and the CME-PEO compound (Fig 3).

**Table 1:** Results of biomechanical analysis in N.

	CONTROL	BURNING	CMC-PEO	LIPOGRAFTING
1	0,19	0,36	0,37	0,32
2	0,24	0,37	0,45	0,27
3	0,25	0,37	0,49	0,35
4	0,26	0,37	0,31	0,3
5	0,28	0,4	0,35	0,26
6	0,29	0,41	0,41	0,4
7	0,31	0,44	0,46	0,31
8	0,32	0,46	0,32	0,34
9	0,32	0,49	0,28	0,5
10	0,32	0,52	0,38	0,38
11	0,34	0,52	0,39	0,43
12	0,37	0,54	0,33	0,29
13	0,38	0,55	0,31	0,35
14	0,38	0,58	0,31	0,34
**MEAN**	0,30	0,46	0,37	0,35
**ST-DEV**	0,06	0,08	0,06	0,07

### Histological analysis

**Fig 4:**
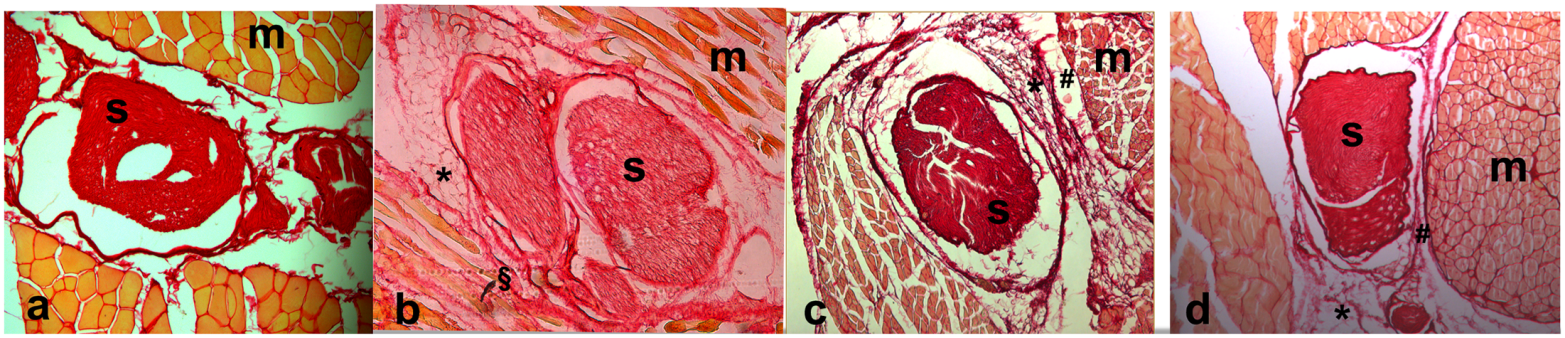
Histological sections of control group (a), burning injury group (b), CMC—PEO group (c) and lipografting group (d). In a the sciatic nerve (S) in normally located between biceps femoris muscles (m). After burning injury (b), the scar tissue (*) between epineurium and epimysium is identifiable and it is possible to see invasion of collagen fibers through muscle fibers (§). After anti-adherence gel (c) or fat graft application (d), scar tissue in yet produced (*), but a gap between muscle and nerve is present (#).

The histological sections are shown in Fig 4. The control group (a) shows the normal relationship between the sciatic nerve (S) and the surrounding muscle (M). After the burning injury, in section b, the pathological scar tissue connects strictly the nerve and the muscle; the layer of collagen fibers (§) is thick and invades the surrounding muscle layer. In the CMC-PEO group (c) there is a tissue gap between the nerve and muscle, despite the presence of the scar tissue. After fat grafting application (d), a collagen layer is still present but with a lower density of fibers; a gap is identifiable between scar tissue and muscles.

## Discussion

The need for preventing perineural adherence after surgery has quickly a series of anti-adherence barriers generally composed of hyaluronic acid, methyl cellulose, ethylen oxide or other synthetic components [22]. The major drawback of these products is the additional cost. Our animal model is an easy and reproducible model to study the perineural adherences and the interaction with anti-adherence devices [18,8]. This study is focused on the validity of the human fat grafting in the prevention of the perineural adherences. The minimal difference in the force that is necessary to minimize the perineural adherence caused by the resistance of newly formed fibrotic tissue between the control and the lipograft group, with similar values compared to CMC-PEO, demonstrate how the adipose tissue can act not only as a mechanical barrier but also as a biological compound with pro-regenerative properties [11, 23, 24].

The idea of using fat grafting to prevent the formation of scar tissue is not new. A similar study of Dumanian et al [25] shows the reduction of scar tissue formation after epineurectomy and mouse fat grafting. However, the model of our study is based on harvesting the fat graft with liposuction technique that has a very low morbidity of the donor site. Therefore, we used human fat in our animals. The authors chose human adipose tissue because of the low quantity of fat tissue that is available. In mice is not possible to lipoaspirate and to centrifuge as in Coleman technique. Moreover, we use of human fat manipulated exactly as in case of a human fat grafting surgery. Some authors [26] proposed fat grafting to prevent capsular contractures after breast implants. Moreover, fat grafting is used in the treatment of Dupuytren disease to prevent a relapse [14]. The use of fat grafting in secondary carpal tunnel release has been recently published [13]. An explanation of its outcome could come from our study. In the event of a secondary operation to carpal tunnel, the addition of the fat graft around the nerve enables it to slide more freely without perineural adhesions.

The fat grafting technique is widely used for treatment after the formation of scar tissue [27] (burn trauma, radiation, cytostatic damage, etc.). In recent years, autologous fat transfer has been used successfully in partial or total reconstruction after mastectomy [28–30] or for aesthetic purposes [31, 32]. The percentage of fat reabsorption was quite low in these studies. Hence, despite the need of further and longer follow up studies, autologous fat transfer may represent a stable anti-adherence compound to prevent perineural scarring.

Lipografting has also gained appreciation in the treatment of post—mastectomy pain syndrome. This would be very interesting to prove in clinical entrapment syndrome where pain is usually the main symptom. [33]

The primary advantages for using an autologous fat grafting as an anti—adherence barrier are the harvesting ease with a quick and simple surgery that can be performed under local anesthesia and the absence of compatibility issues.

Several reasons could explain the efficacy of fat grafting as an anti-adherence barrier. The first and more immediate is the mechanical property of the fat. Lipoaspirate fat is similar to a gel and can easily surround an exposed nerve to protect it from adherence. Secondly, the lipograft is composed by adipocytes but also by SVF (stromal vascular fraction). SVF contains adipose-derived stem cells (ASCs). ASCs are widely known for their pro-regenerative properties [20]. All of these interesting features have made ASCs the main candidate for tissue engineering, including injured peripheral nerves. In particular, ASCs have been used to re-populate de-cellularised nerve grafts used to repair rat nerve gap models [34, 35]. Moreover, after systemic injection of ASCs, few cells have been shown to migrate to the nerve injury site contributing inflammation reduction and nerve regeneration improvement.

According to our results, fat grafting has the anti-adhesion activity similar at already used device in commerce (CMC-PEO) that was hypothesized. Quantitative bio-mechanical analysis shows great anti-adhesion potential for lipografting, confirmed by the descriptive qualitative analysis in which the morphological distribution of collagen fibers is highlighted. The lower adhesion strength of the scar tissue after fat graft application could be explained by the creation of a cleavage plan between the muscle and the nerve. Interestingly, lipografting reduces the production of collagen fibers; this fact is observed in the lower density of the scar layer, but there is no unique reason to explain this fact.

Our findings were derived from a pre-clinical study with limitations. We did not investigate the biochemical mechanisms that drive fat grafting anti-adhesion activity. Thus, further study on animal models will be required and controlled trials on patients will be developed to understand in which cases fat grafting use will be indicated and in which cases a better solution compared to anti-adhesion gel could be used. However, we compare the activity of the graft against anti-adhesion gel that is typically utilized in clinical settings and we find it similar.

## Conclusions

According to our experimental work conducted on athymic mice, fat grafting application around the nerve reduces perineural scar formation, presenting bio-mechanical test values that were comparable compared to synthetic anti-adherence compounds composed of carboxylmethylcellulose with polyethylene oxide (CMC-PEO) molecules.

Considering and confronting the results obtained in this and in another of our previous studies with CMC—PEO compound, we can state that, in an experimental murine model, fat grafting can successfully substitute these anti-adherence compounds.

Thus, we believe that fat grafting can be used with good results as an anti-adherence barrier.
